# Intermittent catheterisation with hydrophilic and non-hydrophilic urinary catheters: systematic literature review and meta-analyses

**DOI:** 10.1186/s12894-016-0191-1

**Published:** 2017-01-10

**Authors:** Carla Rognoni, Rosanna Tarricone

**Affiliations:** 1 Centre for Research on Health and Social Care Management (CERGAS), Bocconi University, Via Roentgen 1, Milan, 20136 Italy; 2 Department of Policy Analysis and Public Management, Bocconi University, Via Roentgen 1, Milan, 20136 Italy

**Keywords:** Intermittent catheterisation, Urinary catheters, Hydrophilic catheters, Urinary tract infections, Haematuria

## Abstract

**Background:**

Intermittent catheterisation is the method of choice for the management of bladder dysfunctions. Different urinary catheters are available, but there is conflicting evidence on which type of catheter is best. The present study provides an objective evaluation of the clinical effectiveness of different subsets of urinary catheters.

**Methods:**

A systematic literature review was performed for published RCTs regarding hydrophilic coated and PVC (standard) catheters for intermittent catheterisation. Separate meta-analyses were conducted to combine data on frequencies of urinary tract infections (UTIs) and haematuria. Two separate analyses were performed, including or excluding reused standard catheters.

**Results:**

Seven studies were eligible for inclusion in the review. The meta-analyses exploring UTI frequencies showed a lower risk ratio associated with hydrophilic catheters in comparison to standard ones (RR = 0.84; 95% CI, 0.75–0.94; *p* = 0.003). Results for the “reuse” scenario were consistent with the ones related to “single-use” scenario in terms of frequency of UTIs. The meta-analyses exploring haematuria were not able to demonstrate any statistically significant difference between hydrophilic catheters in comparison to standard ones.

**Conclusions:**

The findings confirm previously reported benefits of hydrophilic catheters but a broader evaluation that takes into account also patient preferences, compliance of therapy, quality of life and costs would be needed to assess the economic sustainability of these advanced devices.

**Electronic supplementary material:**

The online version of this article (doi:10.1186/s12894-016-0191-1) contains supplementary material, which is available to authorized users.

## Background

Normal bladder functionality can be lost due to neurogenic or non-neurogenic causes. Neurogenic bladder disorders are seen secondary to spinal cord injury (SCI), multiple sclerosis or spina bifida. Common non-neurogenic bladder disorders include outlet obstructions (e.g. benign prostate hyperplasia) and post-operative urinary retention.

Management of bladder dysfunctions aims to improve continence and bladder functionality, protect the upper urinary tract and improve patients’ quality of life. Antimuscarinic agents are the preferred treatment for patients with storage dysfunction, while intermittent catheterisation (IC) is the preferred choice for patients with significant voiding problems [1, 2].

Although antimuscarinic agents are effective, well tolerated and safe, they have no long-lasting therapeutic effects and bladder dysfunction recurs immediately after therapy suspension. As a result, treatment should be continued for the patient’s lifetime. Available alternative treatments include intra-detrusor injection of botulinum toxin and neuromodulation. Botulinum toxin causes a reversible chemical denervation, lasting for approximately 9 months, which can significantly improve bladder functionality. Electrical stimulation of peripheral nerves (e.g. sacral or pudendal nerve), interrupting inappropriate detrusor contractions, has proved to be effective in managing the idiopathic overactive bladder [3], but for patients with an underlying neurological disorder, its role still remains unclear [1].

In patients with bladder storage dysfunction, urinary catheterisation can be required in combination with antimuscarinic agents, botulinum toxin or neuromodulation if voiding problems occur. Incomplete bladder emptying can either be managed by a permanent urethral/suprapubic catheter or IC. European guidelines focusing on neurogenic lower urinary tract dysfunctions [4–6] report that IC is the option of choice for patients resulting in high post-void residual volumes, especially for patients with SCI [7]. IC is a manual bladder emptying technique performed regularly about four to six times a day by a patient themselves or a caregiver; the catheter is inserted through the urethra and removed once the bladder has been drained from urine. This method limits the complications and improves the prognosis of the patients [8].

One of the major advantages of IC is the significant reduction in the risk of catheter-induced UTIs, resulting in maintenance of urinary tract health and protection of the kidneys [9, 10]. In 2010 the International Consultation on Incontinence concluded that IC is effective and safe for emptying the bladder both in the short and long terms, but that bladder and urethral complications increase in the long term [11]. These complications are mainly represented by recurrent UTIs, which are one of the most important problems of patients with lower urinary tract dysfunction. These infections, if not treated properly, can lead to kidney infections, resulting in kidney failure and risk of sepsis [12]. UTIs also cause high morbidity and result in frequent hospitalisations [13]. Moreover, repeated cycles of antibiotic therapy necessary in patients with a recurrent UTI cause the onset of “antibiotic resistance” in various strains of microorganisms involved in the infection [12]. For these reasons, UTIs impose in general a relevant economic burden on patients and their families as well as on the healthcare systems [14].

IC performed several times a day places the individual at risk also for urethral trauma, often measured by the occurrence of haematuria. Urethral trauma is associated with an increase in UTI risk [15, 16].

There are different catheters suitable for IC, for example, disposable catheters with a hydrophilic polymer surface coating, disposable catheters with pre-packaged water based lubricant, and uncoated catheters. Uncoated catheters may be discarded after use or washed and re-used for different days.

Two possible advantages of hydrophilic coated catheters over uncoated ones are the reduction of urethral trauma (e.g., haematuria) and the incidence of symptomatic UTIs. Currently, although there are trends in favour of hydrophilic coated catheters with respect to UTIs [17–19] in the short term, there is little consensus on which type of catheter is best. Four meta-analyses have been previously published investigating the impact of hydrophilic coated catheters (and other catheter types) on UTI rate and urethral trauma among patients practicing IC [20–23]. Two meta-analyses concluded that hydrophilic coated catheters are associated with a risk reduction of UTI [20, 23] and trauma [23] as compared to non-hydrophilic catheters, while two others were inconclusive and unable to differentiate between catheter types or techniques [21, 22]. According to Clark et al. [20] the effect size of UTI reduction were 21% in hospital setting and 53% in the long-term community setting [20] while Li et al. [23] reported a risk reduction of 64% [23] associated to hydrophilic coated catheters. The other two meta-analyses [21, 22] allowed more catheter types in the comparison and as such the included studies were more heterogeneous and, accordingly, they showed no treatment difference between catheter types or catheter techniques in terms of UTI rate. In addition, they concluded that the number of randomised controlled trials were too low and compromised by quality issues [21, 22]. Two studies added cost-effectiveness data based on the results of the meta-analyses [20, 22]; one study concluded that hydrophilic coated catheters is a cost-effective choice when considering long-term treatment of IC [20] while the other one concluded that there are no therapy or economic benefits associated to a specific catheter type or technique [22].

The aim of the present study was to confirm/reject the conflicting evidence of previously published meta-analyses and again try to compare complication rates (UTI and urethral trauma/haematuria) related to hydrophilic coated catheters as compared to non-hydrophilic catheters for users who practice IC. In addition, a separate analysis including reused uncoated catheters was performed to evaluate differences between catheter types and their use in IC.

## Methods

### Literature search

The present review adopts the Preferred Reporting Items for Systematic Reviews and Meta-Analysis (PRISMA) statement [24]. In June 2016 a systematic search was conducted on MEDLINE/PUBMED, EMBASE, the Cochrane Library, and Web of Science databases to retrieve clinical evidence. The search strategy was developed using the PICO (Patient, Intervention, Comparator, Outcome) Study framework.

Boolean operators “AND” and “OR” were used to combine terms while the “NOT” operator, following Cochrane indications, was not included.

Studies were considered if published in English and if they referred to an adult or adolescent population. Studies were included provided that they directly compared the use of the two devices on clinical evidence. Both single-use and re-used catheters were considered. Case reports, letters, comments, editorials, and non-systematic review were excluded.

### Selection criteria

Inclusion criteria are shown in Table 1 (see Appendix for detailed search query).

**Table 1:** PICO inclusion criteria

Population	Studies considering adult or adolescent population with bladder dysfunctions requiring IC
Intervention	Hydrophilic catheters – single-use
Comparator	Non-hydrophilic catheters – single-use or multiple-use
Outcome	UTIs, haematuria
Study	Randomised controlled trials or randomised cross-over trials
Availability	English; full text
Time and place	Date and place limits were not set for this review

### Data extraction

Abstracts and full-text selection was conducted independently by two expert reviewers (CR, RT). In case of debate on eligibility, studies were verified collaboratively until a consensus was obtained. Clinical data were extracted using a customised template developed in Microsoft Excel, including study features, participants’ characteristics, and clinical outcomes. Studies considering single-use catheters have been separated from the ones considering reused catheters.

### Data analysis

Clinical data directly comparing hydrophilic and non-hydrophilic catheters were considered for meta-analysis. The meta-analysis focused on two clinical outcomes: symptomatic UTIs and haematuria (bleeding episodes). For symptomatic UTIs we mainly referred to the definition supplied by the National Institute on Disability and Rehabilitation Research [25]: positive urine culture with pyuria and one or more systemic symptoms as fever, loin pain, dysuria, urgency, haematuria. In any case, we also evaluated studies reporting symptomatic UTIs according to other definitions or studies where an exact definition for symptomatic UTI was not provided. As regards haematuria, we considered the following definitions: presence of red blood cells in the urine, urethral bleeding, gross haematuria. Studies reporting microscopic haematuria were also considered.

Separate meta-analyses were conducted to combine the results of the retrieved studies on relative risk (RR) of developing UTIs and haematuria using Review Manager (RevMan5) software (Version 5.1. Nordic Cochrane Centre, Cochrane Collaboration, 2011. http://community.cochrane.org/tools/review-production-tools/revman-5).

Since the considered studies were performed by researchers working independently, a random-effect model was applied assuming that the true effect size varies from one study to the other [26]. A test on the summary effect measure is given, as well as a test for heterogeneity quantified by I^2^ (range 0–100%). Higher values of it represent higher heterogeneity among the studies [27].

Results are displayed in forest plots according to different catheter subgroups and employments.

### Quality assessment

The evaluation of potential biases in the selected studies is an essential element of a systematic literature review or meta-analysis. The methodological quality of included studies was assessed according to the Cochrane Collaboration’s Risk of Bias tool in Review Manager software (RevMan 5 - http://community.cochrane.org/tools/review-production-tools/revman-5). Following CRD guidance [28], no scoring system was adopted; rather, quality assessments were used for descriptive purposes. The risk of bias assessment was performed in the following domains: sequence generation; allocation concealment; blinding of participants and personnel and outcome assessors; blinding of outcome assessment; incomplete outcome data; selective outcome reporting.

## Results

### Literature search

**Fig. 1:**
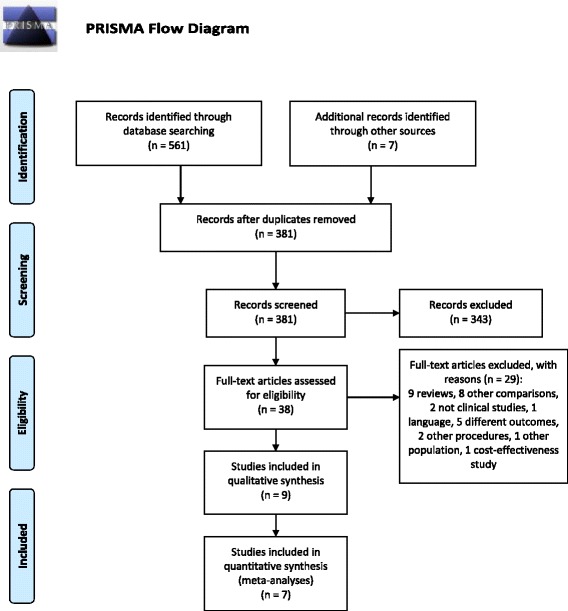
Study selection process

A total of 561 studies were found through PUBMED (197), EMBASE (19), Web of Science (202) and Cochrane (143). After the removal of duplicates (180), 381 studies were obtained. Reference lists of the most relevant retrieved articles were screened to find additional studies (7) not identified through the initial database search. Figure 1 shows the selection process and reasons for the exclusion of studies at each step, while Table 2 shows the nine studies eligible for the final evaluation with their characteristics (studies included in quantitative synthesis are marked with ‘*’).

**Table 2:** Summary of extracted clinical data

Study	Type	Population	Study duration	Hydrophilic Arm (H)			Non-hydrophilic arm (NH)			UTIs	Haematuria (number of patients or episodes)
				Patients	Mean age	Catheter brand/type	Patients	Mean age	Catheter brand/type		
Single-use standard catheters											
Cardenas 2009 [17] *	RCT	Neurological	12 months	22 (77% males)	42.3 ± 10.4	LoFric/NA	23 (52% males)	40.1 ± 9.3	NA/NA	Mean number of UTIs: H: 1.18 ± 1.3 NH: 1.00 ± 1.0 (*p* = NS) Subjects who had at least one UTI: H: 12 (54%) NH: 14 (61%) (*p* = NS)	NA
Cardenas 2011 [18] *	RCT	Neurological	6 months	105 (79% males)	37.2 ± 14.4	Coloplast/Speedicath	114 (82% males)	35.1 ± 13.2	Coloplast/Conveen	Mean number of UTIs per month (according to clinical definition): *Full study period* H: 0.479 NH: 0.478 (*p* = NS) *Institutional period* H: 0.539 NH: 0.682 (*p* = 0.038) Mean number of UTIs per month (according to strict definition): *Full study period* H: 0.198 NH: 0.218 (*p* = NS) *Institutional period* H: 0.189 NH: 0.295 (*p* = 0.022) Subjects who had at least 1 UTI (estimated from curves): H: 69 out of 91 patients NH: 85 out of 97 patients	H: 14 NH: 6 (*p* < 0.0001)
De Ridder 2005 [19] *	RCT	Neurological	12 months	61 (100% males)	37. 5 ± 14.6	Coloplast/Speedicath	62 (100% males)	36.7 ± 14.6	Coloplast/Conveen	Subjects who had at least one UTI: H: 39 (64%) NH: 51 (82%) (*p* = 0.02)	H: 38 out of 55 patients NH: 32 out of 59 patients (*p* = NS)
Massa 2009 [29]	RCT	Neurological	12 months	25	NA	LoFric/NA	26	NA	NA/NA	Number of UTIs in the first 3 months: H: 17 NH: 12 (*p* = NS)	NA
Sarica 2010 [30] *	Crossover	Neurological	18 weeks	10 (100% males)	37.04 ± 11.86	Teleflex/Rusch Flocath	10 (100% males)	37.04 ± 11.86	NA/NA	Subjects who had at least one UTI: H: 1 NH: 4 (*p* = NS)	NA
Wyndaele 2000 [31] *	Crossover	Neurological	2 years	39 (100% males)	45 ± 15	Coloplast/UroCath Gel	39 (100% males)	45 ± 15	NA/NA	NA	H: 10 NH: 9 (*p* = NS)
Reused standard catheters											
Pachler 1999 [33] *	Crossover	Non-neurological	3 weeks	32 (100% males)	71.3 years (range 50–87)	LoFric/NA	32 (100% males)	71.3 years (range 50–87)	Mentor/NA	Subjects who had at least one UTI: H: 2 NH: 2 (*p* = NS)	H: 2 NH: 2 (*p* = NS)
Sutherland 1996 [34] *	RCT	Neurological or non-neurological	8 weeks	17 (100% males)	11.7 ± 3.8	LoFric/NA	16 (100% males)	12.1 ± 5.7	Mentor/NA	Subjects who had at least 1 UTI (bacteriuria and fever): H: one out of 16 patients NH: one out of 14 patients (*p* = NS)	H: six out of 16 patients NH: 11 out of 14 patients
Vapnek 2003 [32]	RCT	Neurological	12 months	30 (100% males)	39.8 ± 12.9	LoFric/NA	31 (100% males)	39.6 ± 16.0	NA/NA	Mean number of UTIs per month per patient: H: 0.13 ± 0.18 NH: 0.14 ± 0.21	NA

* = included in meta-analysis, *H* = hydrophilic coated catheter, *NH* = non-hydrophilic coated catheter, *RCT* = randomised clinical trial, *NS* = not significant, *NA* = not available

Most studies included a population with neurological disorders. Cardenas and colleagues considered both patients with SCI occurred at least 6 months [17] and less than 3 months [18] before study inclusion. Also other studies included patients with neurogenic bladder due to SCI [19, 29, 30]. Other two studies [31, 32] included patients with neurogenic bladder without specifying the origin. Pachler and colleagues [33] considered males with urinary retention due to prostatic enlargement, while Sutherland et al. [34] involved in the study boys with voiding dysfunctions due to different causes (spinal dysraphism, spinal cord injury or Hinman syndrome).

### Meta-analysis results

The first meta-analysis was performed on the subset of studies considering single-use of both hydrophilic catheters and the comparators. Other studies referred to a control group of PVC catheters used generally 4–5 times per day and then discarded (i.e. reuse). After each use, the catheter is rinsed under lukewarm water and left to dry on a clean towel. Since there is a lack of evidence about the impact of single- or multiple-use catheters on the incidence of UTIs and haematuria [21], two separate meta-analyses were carried out, one including only single-use control and one including all available studies.

#### a) Single-use only sub-analysis

Three trials reported the number of patients with at least one symptomatic UTI [17, 19, 30]. Among them, one study [30] was designed to compare the use of standard polyvinyl chloride, hydrophilic-coated, and gel-lubricated non-hydrophilic catheters and only data related to the first two items were used due to the pre-defined aim of the study.

One study [18] reported the total number of UTIs for both groups for the full study period for both strict and clinical definitions of UTI. These data couldn’t be used since there was no indication on the number of patients experiencing UTIs. On the other hand, the percentages of patients experiencing at least one UTI were retrieved by digitalising (TechDig software) figures reporting the time from the first catheterisation to the onset of the first symptomatic UTI for both devices. From that figure, percentages of patients reporting UTIs of 75 and 87 were estimated respectively for the use of hydrophilic and non-hydrophilic catheters.

Another study [29] refers to the same population of [17] and was excluded from the analysis. Moreover, this study reported only the total number of UTIs per type of catheter that were not usable for this analysis.

**Fig. 2:**
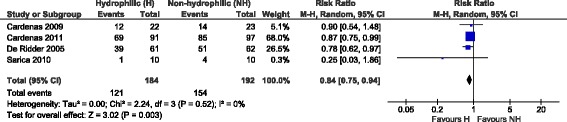
Meta-analysis results related to UTIs (single-use catheters)

The meta-analysis results are reported in Fig. 2. The estimate from these trials highlights a statistically significant decreased risk ratio of UTIs associated with hydrophilic catheters in comparison with non-hydrophilic ones (RR = 0.84; 95% CI, 0.75–0.94; *p* = 0.003). There was no evidence of significant heterogeneity across the studies (*p* = 0.52).

**Fig. 3:**
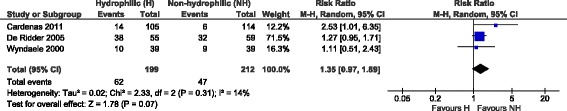
Meta-analysis results related to haematuria (single-use catheters)

Three trials reported the number of patients experiencing episodes of haematuria or urethral bleeding [18, 19, 31]. The incidence of haematuria was 31% (62/199) in patients using hydrophilic catheters and 22% (47/212) in patients using non-hydrophilic catheters. Although not statistically significant, the estimate from these trials point to a higher risk of developing haematuria with hydrophilic catheters versus the standard ones (RR = 1.35; 95% CI, 0.97–1.89; *p* = 0.07). This analysis didn’t report a significant heterogeneity among the considered studies (Fig. 3).

#### b) Single-use/multiple-use sub-analysis

A second step meta-analysis including all the available evidence (i.e. also studies with a control group where non-hydrophilic catheters were reused).

In one study [32], the number of patients experiencing at least one UTI or haematuria was not estimable due to how the data were described. As to UTIs, only the mean number per patient was reported, while the degree of haematuria was classified as none, mild, moderate, and heavy, and to each category a code from 0 to 3 was assigned. The mean code per urinalysis during the study period was reported.

Only the results reported by [33] and [34] were added for the second step meta-analysis.

**Fig. 4:**
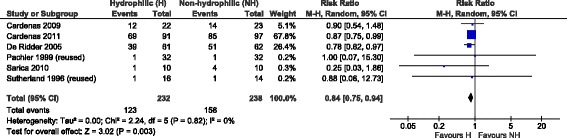
Meta-analysis results related to UTIs (single- and multiple-use catheters)

The results on UTIs (Fig. 4) showed the same results as the initial analysis performed considering only single-use in the control group, i.e. a risk reduction associated to hydrophilic coated catheters was verified.

**Fig. 5:**
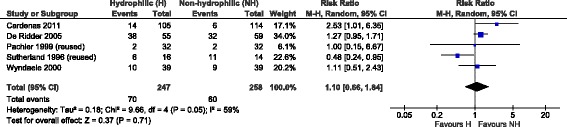
Meta-analysis results related to haematuria (single- and multiple-use catheters)

The results from the two meta-analyses on haematuria (Figs. 3 and 5) differed slightly and the added reuse-studies seemed to reduce the risk of experiencing haematuria.

### Quality assessment

In the studies, it was not possible to blind participants, but the non-blinding of participants was considered unlikely to introduce bias in six of them. Three trials [17, 19, 30] reported high percentages of dropouts that were more frequent in the hydrophilic catheter arm, thus resulting in an imbalance and in potentially biased results. In particular, only 43% vs. 60% and 41% vs. 53% of patients in the hydrophilic and non-hydrophilic arms remained in the study respectively for [17] and [19] studies. Sarica and colleagues [30] reported missing data from 15 participants on a total of 25 on one or more catheters. The assessment of potential biases for the considered clinical studies is reported in Additional file 1: Figure S1 and Additional file 2: Figure S2.

## Discussion

The aim of the present study was to confirm or reject the conflicting evidence of previously published meta-analyses [20–23] and again try to evaluate complication rates (UTI and urethral trauma/haematuria) related to hydrophilic coated catheters as compared to non-hydrophilic catheters for users who practice IC. In addition, a separate analysis including reused uncoated catheters was included to evaluate the possibility to further differentiate between catheter types and their use in IC.

The results from the study showed that hydrophilic coated catheters are associated with a reduced risk of UTIs among patients performing IC. The estimated risk reduction was 16% considering both single-use and single-use plus reused catheters scenarios. No difference in the results is due to the low number of patients involved in the two additional studies considering reused devices, which accounted for low study weights (0.2% each) in the overall analysis.

As regards the second considered outcome, haematuria, the meta-analyses were not able to verify a risk reduction associated to hydrophilic coated catheters. However, the results from the two meta-analyses suggest that there may be differences related to types of hydrophilic coated catheters. It should be noted that hydrophilic catheters considered in the single-use scenario all referred to the same brand (i.e. Coloplast), while both additional studies included to consider the extended scenario referred to another brand (i.e. LoFric). The inclusion in the analysis of these hydrophilic coated catheters with high osmolality [35] seemed to lower the risk of haematuria, although statistical significance could not be verified.

The present study provided objective data to support the use of hydrophilic catheters in clinical practice to reduce UTIs; however, the opinion of the patient regarding the choice of the type of device should also be taken into account; he/she has to find the product agreeable, corresponding to his/her needs, handy, and easy to use.

The present review has some limitations, first of all, the heterogeneity regarding the clinical outcomes and their definitions in the included studies. The proposed definitions of symptomatic UTI were: significant bacteriuria (≥10^5^ CFU/mL) plus at least one sign or symptom suggestive of UTI [17], clinical definition of symptomatic UTI (antibiotic treatment prescribed), and strict definition of symptomatic UTI (antibiotic treatment prescribed, bacteriuria, at least one of seven symptoms based on consensus guidelines–fever, autonomic dysreflexia, increased spasticity, discomfort or pain over the kidney or bladder or during micturition, onset and/or increase in incontinence episodes, cloudy urine with increased odour, malaise, lethargy, or sense of unease; dipstick test positive for leukocyte esterase) [18], clinical infection with symptoms of UTI and for which treatment was prescribed [19], >10^4^ CFU/mL [33], infection of the urinary tract that requires the insertion of a Foley catheter [30], >10^5^ CFU/mL [34]. With regard to haematuria, no precise definition was given but the studies referred to microscopic haematuria [34], gross haematuria [33], urethral bleeding [18, 31] and haematuria in general [19, 32].

Secondly, nearly half of the trials presented attrition biases that can greatly influence the strength of the reported results. Moreover, dropouts occurred early and were more frequent in the arm related to hydrophilic catheters, thus resulting in an imbalance and a potential bias in favour of the latter. This means that patients who didn’t continue the study may have been less satisfied with hydrophilic catheters than those who completed the study.

Thirdly, effectiveness data were derived from few RCTs with less than 50 participants. Although systematic reviews can be performed in practice with any number of studies, when few studies are used, the heterogeneity point estimate I^2^ should be interpreted cautiously, even replaced with confidence intervals as reported by von Hippel [36].

Another limitation of the current study is that UTIs and episodes of haematuria are not the only complications that can occur in users performing ICs, However, the former are the most frequent complications in this type of users, while the latter occur regularly in one-third of them on a long-term basis [37].

In spite of the limitations of the current review and meta-analyses, the results from two previously published reviews [20, 23] in terms of UTI risk reduction associated to the use of hydrophilic coated catheters were verified. It should be noted that the meta-analyses of this study were limited to randomised clinical trials only to ensure high level of evidence but this is a limitation per se since few high quality trials exist and the available ones are compromised by quality issues [21]. On the other hand, the results from this study also verify the results by several observational studies that focused on the frequencies of UTIs [38, 39], urethral trauma [39], urethral complications [38], microscopic haematuria, and pain [40].

The management of UTIs with systemic symptoms requiring medical intervention is associated with significant costs. Findings can be summarised by a wide cost span between €523 and €4167 [41–46] and it is likely that more complicated UTIs are associated with higher costs. A catheter that could lower UTI frequencies and other types of complications is likely to limit the burden for patients using IC, resulting in increased quality of life. The combination of both economic and quality of life aspects can be evaluated through a cost-effectiveness analysis comparing hydrophilic catheters to non-hydrophilic catheters.

## Conclusions

The meta-analyses results confirmed that hydrophilic coated catheters are associated with a reduced risk of UTI among patients using IC. On the other hand, a risk reduction for haematuria associated to hydrophilic coated catheters in general was not demonstrated. The conclusions from the study are however compromised by several limitations, such as the heterogeneity of outcomes and definitions, the lack of available high quality randomised controlled trials as well as a higher dropout rate in the arms related to hydrophilic catheters. In view of these limitations, uncoated catheters may still maintain a place in the clinical practice.

Further studies are crucial to provide more direct evidence of the comparison between hydrophilic versus non-hydrophilic coated catheters and could be used to integrate a cost-effectiveness model. In the meantime, it is important also to consider the evidence from observational data when assessing the effectiveness of hydrophilic-coated catheters.

In conclusion, there is still further work to be performed in order to assess incremental cost and effectiveness of hydrophilic versus standard catheters to optimise informed policy decisions.

## Appendix

SEARCH QUERY:

- *(spinal OR SCI OR SCIs OR neurogenic OR bladder OR urinary OR urethral OR dysfunction)*
- *AND*
- *(hydrophilic OR LoFric OR coated OR POBE OR polyolefin based elastomer OR polyolefin-based elastomer OR PVC free OR PVC-free OR Speedicath OR Easicath)*
- *AND*
- *(standard OR conventional OR plastic OR polyethylene OR PVC OR polyvinyl OR nonhydrophilic OR non hydrophilic OR non-hydrophilic OR non coated OR non-coated)*
- *AND*
- *(intermittent OR catheter*)*
- *AND*
- *(urinary tract infection* OR UTI OR UTIs OR infection* OR urethral trauma OR stricture* OR hematuria OR haematuria)*

## Additional files

Additional file 1: Figure S1. Risk of bias summary; Judgments regarding risks of bias for each study included in the systematic review. (PDF 211 kb)
Additional file 2: Figure S2. Risk of bias graph; Judgments regarding risks of bias presented as percentages across all studies included in the systematic review. (PDF 206 kb)

## Abbreviations

H: Hydrophilic coated catheters
IC: Intermittent catheterisation
NA: Not available
NH: Non-hydrophilic coated catheters
PVC: Polyvinyl chloride
RCT: Randomised controlled trial
RR: Relative risk
SCI: Spinal cord injury
UTI: Urinary tract infection
