# Supplementary material for: Intermittent catheterisation with hydrophilic and non-hydrophilic urinary catheters: systematic literature review and meta-analyses
Source: BMC Urol. 2017 Jan 10;17:4. doi: 10.1186/s12894-016-0191-1 (PMC5225586; doi:10.1186/s12894-016-0191-1)
Supplement: Additional file 1: Figure S1. — Risk of bias summary; Judgments regarding risks of bias for each study included in the systematic review. (PDF 211 kb) [file 12894_2016_191_MOESM1_ESM.pdf]

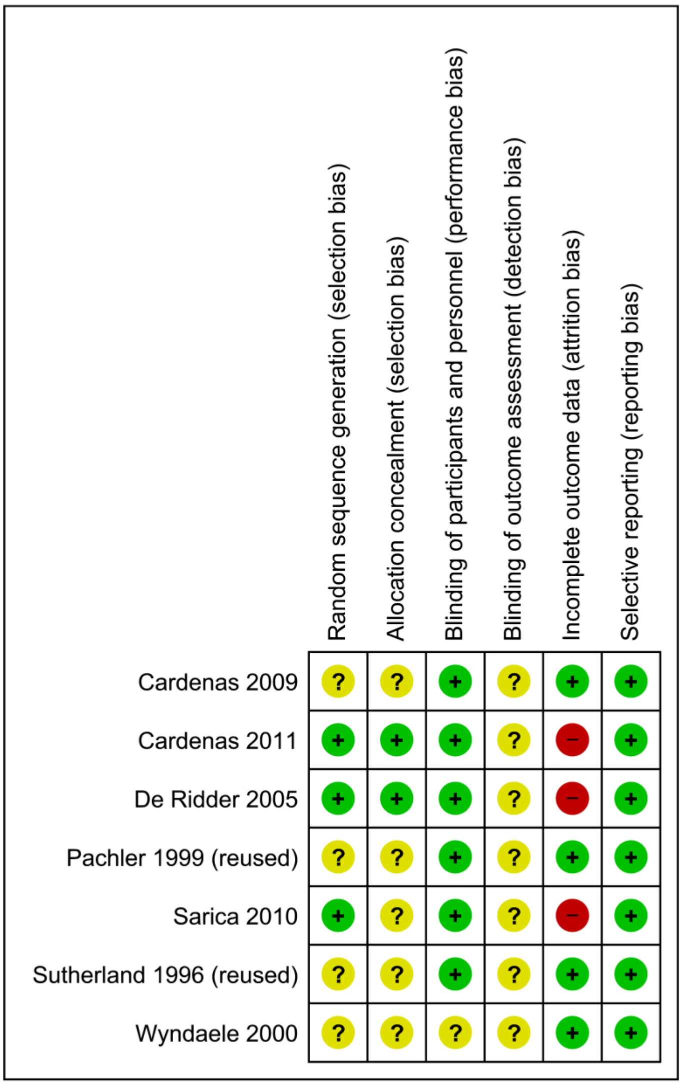

Supplementary Figure 1 – Risk of bias summary: judgments regarding risks of bias for each study included in the systematic review (n=7). The symbol "+" represents low risk of bias, the symbol "?" represents unclear risk of bias and the symbol "-" represents high risk of bias.
