# Supplementary material for: Intermittent catheterisation with hydrophilic and non-hydrophilic urinary catheters: systematic literature review and meta-analyses
Source: BMC Urol. 2017 Jan 10;17:4. doi: 10.1186/s12894-016-0191-1 (PMC5225586; doi:10.1186/s12894-016-0191-1)
Supplement: Additional file 2: Figure S2. — Risk of bias graph; Judgments regarding risks of bias presented as percentages across all studies included in the systematic review. (PDF 206 kb) [file 12894_2016_191_MOESM2_ESM.pdf]

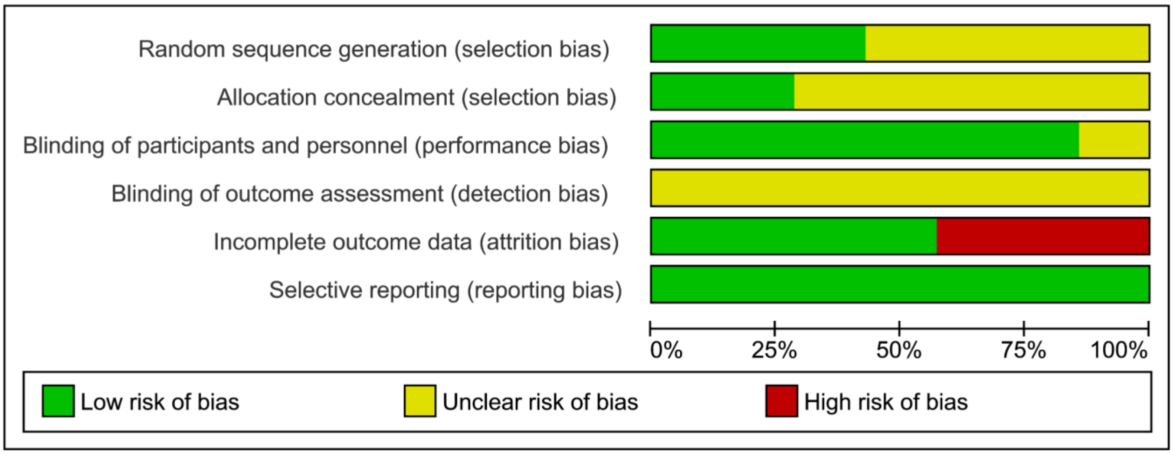

Supplementary Figure 2 – Risk of bias graph: judgments regarding risks of bias presented as percentages across all studies included in the systematic review (n=7). This figure illustrates, for each considered bias domain, the proportion of studies falling in each category of risk (low risk of bias, high risk of bias, unclear risk of bias).
